# Supplementary material for: Neuronal processes and glial precursors form a scaffold for wiring the developing mouse cochlea
Source: Nat Commun. 2020 Nov 17;11:5866. doi: 10.1038/s41467-020-19521-2 (PMC7672226; doi:10.1038/s41467-020-19521-2)
Supplement: Supplementary file 1 — Supplementary Information [file 41467_2020_19521_MOESM1_ESM.pdf]

Supplementary Information

This document includes two supplementary figures and a supplementary table for the paper entitled "Neuronal processes and glial precursors form a scaffold for wiring the developing mouse cochlea" by Druckenbrod et al.

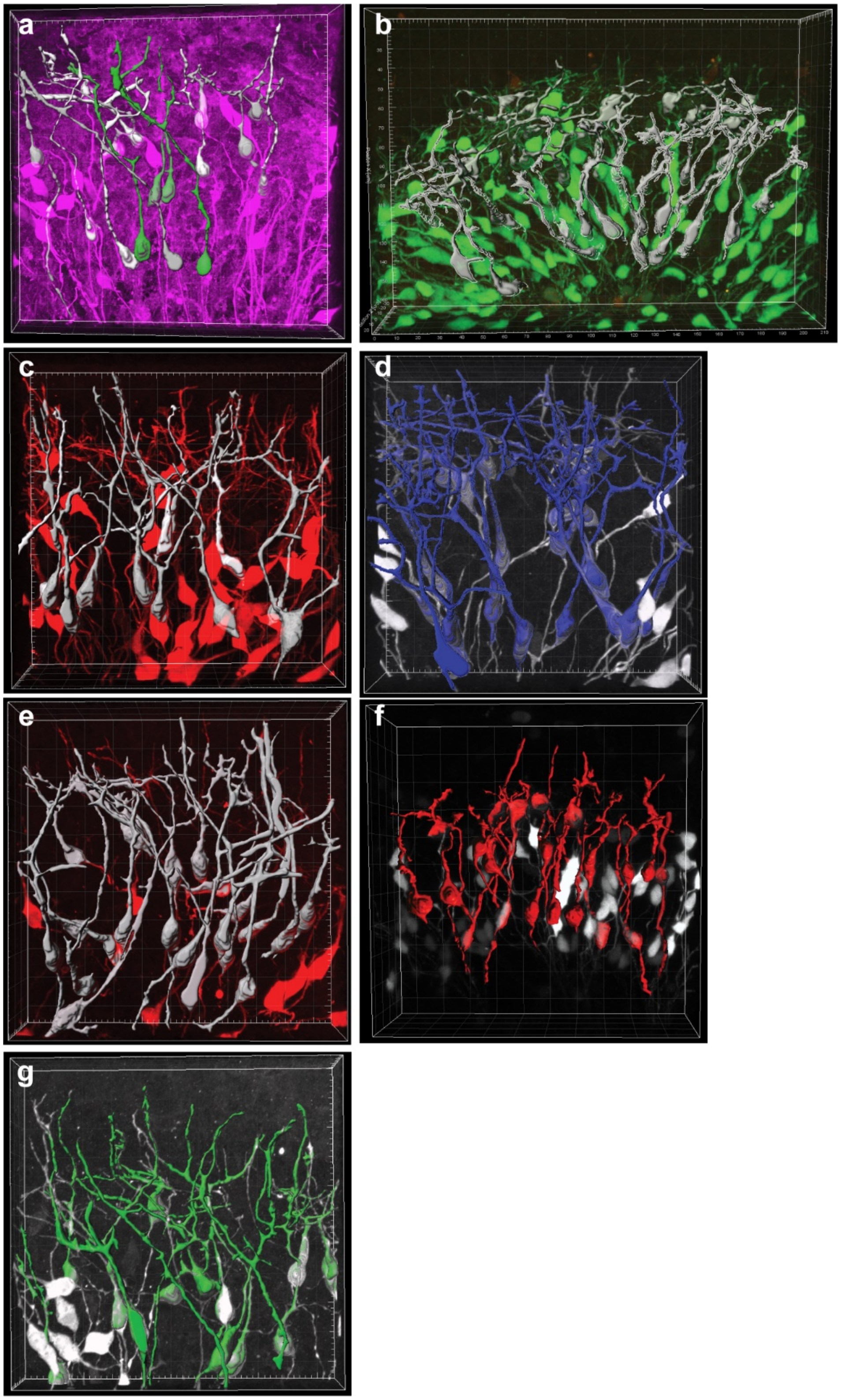

Supplementary Figure 1: Spiral Ganglion Neuron Reconstructions

(**a-g**) Projections of confocal z-stacks through the regions of *Neurog1^{CreERT2};Ai14* cochleae (N=7) that were used to generate the data shown in Figure 2. The tdTomato signal was enhanced by anti-DsRed immunostaining. The overall pattern of SGN staining is shown in magenta (a), red (c, e), green (b), or in white (d, f, g). Reconstructions are superimposed on the staining and colored gray (a-c, e), green (a,g), blue (d), or red (f). A total of 151 SGNs were reconstructed and analyzed.

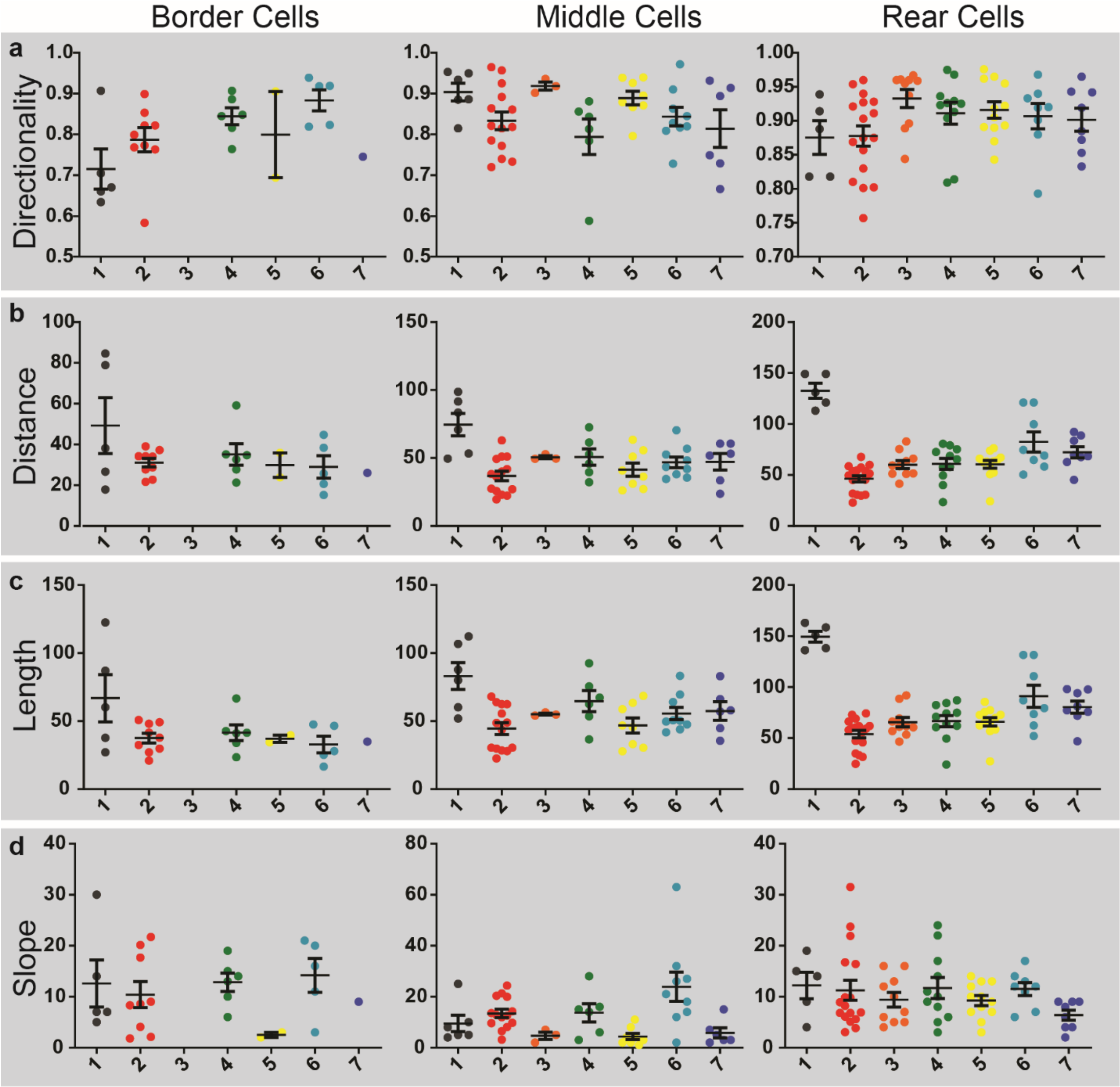

Supplementary Figure 2: Quantification of SGN morphology in individual cochleae

(**a-d**) Metrics from the seven individual cochleae used to generate data in Figure 2, with cochleae 1-7 corresponding to Supp Fig. 1a-g. For each cochlea, directionality (a), distance (b), length (c), and slope (d) were calculated for cells sitting in the border, middle or rear regions. Note that the axes change for each population so that all of the individual data points can be seen easily. N= 151 total cells from 7 cochleae. Mean and standard error of mean are shown for each population.

Figure	Description	N/n	Test	*P*-value Results
1i	Percentage of wavefront SGN processes preceded by glial precursors in mid-base of fixed cochlea	Tuj control:<br>7 cochleae; 164 neurites<br>Neurog1^{CreERT2} control:<br>6 cochleae; 125 neurites	Unpaired t-test (two-tailed)	Tuj Control v Neurog1^{CreERT2} Control:<br>P=0.5692
2c	Inter-ganglion border, middle, and rear: SGN process directionality comparison	7 cochleae; n=28 border cells; 52 middle cells; and 71 rear cells	Normality test; ANOVA with Tukey's multiple comparison test	Border v Middle: P=0.0188<br>Border v Rear: P<0.0001<br>Middle v Rear: P=0.0003
2d	Inter-ganglion border, middle, and rear: SGN process length comparison	7 cochleae; n=28 border cells; 52 middle cells; and 71 rear cells	Normality test; One-way ANOVA with Tukey's multiple comparison test	Border v Middle: P=0.0025<br>Border v Rear: P<0.0001<br>Middle v Rear: P<0.0001
2e	Inter-ganglion border, middle, and rear: SGN process slope comparison	7 cochleae; n=28 border cells; 52 middle cells; and 71 rear cells	Normality test; One-way ANOVA with Tukey's multiple comparison test	Border v Middle: P=0.107<br>Border v Rear: P=0.0002<br>Middle v Rear: P=0.0384
3d	R1-3 process speed comparison	Cochleae/region: 3<br>Processes/region:<br>55 (R1), 64 (R2), 44 (R3)	Normality test; ANOVA with Tukey's multiple comparison test	R1 v R2: P<0.0001<br>R1 v R3: P=0.6107<br>R2 v R3: P<0.0001
3e	R1-3 process directionality comparison	Cochleae/region: 3<br>Processes/region:<br>55 (R1), 64 (R2), 44 (R3)	Normality test; ANOVA with Tukey's multiple comparison test	R1 v R2: P<0.0001<br>R1 v R3: P=0.9782<br>R2 v R3: P<0.0001
4b	Sparsely labelled SGN directionality in R1: inter-regional comparison	Cochleae: 3<br>25 Border Cells<br>24 Rear Cells	Unpaired t-test (two-tailed)	R1 Border v Rear cell: P<0.0001
4d	Sparsely labelled SGN directionality in R2: inter-regional comparison	Cochleae: 3<br>Wavefront: 17<br>Behind: 23	Unpaired t-test (two-tailed)	R2 Border v Rear cell: P<0.0001
5b	SGN process behavior: comparison of directionality ON or OFF glial precursors	4 cochleae; 35 processes	Unpaired t-test (two-tailed)	ON vs OFF-glia: P<0.0001
5c	SGN process behavior: comparison of speed ON or OFF glial precursors	4 cochleae; 35 processes	Unpaired t-test (two-tailed)	ON vs OFF-glia:P= 0.0428

Supplementary Table 1: Statistical summary

A summary of the group sizes and statistical tests used to analyze data presented in this manuscript.
