# Supplementary material for: Neuronal processes and glial precursors form a scaffold for wiring the developing mouse cochlea
Source: Nat Commun. 2020 Nov 17;11:5866. doi: 10.1038/s41467-020-19521-2 (PMC7672226; doi:10.1038/s41467-020-19521-2)
Supplement: Supplementary file 3 — Description of Additional Supplementary Files [file 41467_2020_19521_MOESM3_ESM.pdf]

Description of Additional Supplementary Files

Title: Supplementary Movie 1

Description: Heterogeneous SGN morphology in the E14 ganglion. A video illustrating a typical set of reconstructed SGNs in the ~E14 Neurog1CreERT2; Ai14 cochlea. Rear SGNs are shown in purple; middle SGNs in green; and border SGNs in yellow. The same SGNs are shown in Fig. 2b.

Title: Supplementary Movie 2

Description: SGN peripheral process outgrowth in R1. An example of SGN peripheral process behavior at the wavefront of early outgrowth, as imaged in a ~E14.25 Bhlhb5Cre; Ai14 cochlea. This region corresponds to R1, as illustrated in Fig. 3a, b. Frames from this movie are shown in Fig. 3g.

Title: Supplementary Movie 3

Description: SGN peripheral process outgrowth in R2. A representative example of SGN peripheral process behavior in an E15 Bhlhb5Cre; Ai14 cochlea. By this time, the wavefront of outgrowth has reached R2, as illustrated in Fig. 3a, b.

Title: Supplementary Movie 4

Description: SGN peripheral process outgrowth in R3. A representative example of SGN peripheral process behavior in an E15.5 Bhlhb5Cre; Ai14 cochlea. By E15.5, peripheral processes have reached R3, where the organ of Corti is developing, as illustrated in Fig. 3a, b.

Title: Supplementary Movie 5

Description: Individual SGN processes follow distinct trajectories in R1. A movie of SGN peripheral process behavior in R1 of an E14.25 Neurog1CreERT2; Ai14 cochlea. In this case, a random subset of SGNs are fluorescently labeled, making it possible to distinguish processes from SGNs whose cell bodies sit at the border from those whose cell bodies are further behind and out of view (movement of the growth cone is indicated by arrows). Frames from this movie are shown in Fig. 4a.

Title: Supplementary Movie 6

Description: Individual SGN processes exhibit distinct outgrowth behaviors in R2. A movie of SGN peripheral process behavior in R2 of an ~E15 Neurog1CreERT2; Ai14 cochlea. By this stage, the

processes at the wavefront of growth show more exploratory behavior than those that start from further behind. Frames from this movie are shown in Fig. 4c.

Title: Supplementary Movie 7

Description: Neuron-glia precursor interactions in the developing cochlea. A movie from a ~E14-E14.5 Neurog1CreERT2; Ai14; PLP-GFP cochlea, with tdTomato+ SGN peripheral processes in red and PLP-GFP+ glial precursors in green. This movie highlights behavior at the wavefront. Frames from this movie are shown in Fig. 5d.

Title: Supplementary Movie 8

Description: Neuron-glia precursor interactions in the developing cochlea. A movie from a ~E14-E14.5 Neurog1CreERT2; Ai14; PLP-GFP cochlea, with tdTomato+ SGN peripheral processes in red and PLP-GFP+ glial precursors in green. This movie highlights behavior at the wavefront.

Title: Supplementary Movie 9

Description: Neuron-glia precursor interactions in the developing cochlea. A movie from a ~E14-E14.5 Neurog1CreERT2; Ai14; PLP-GFP cochlea, with tdTomato+ SGN peripheral processes in red and PLP-GFP+ glial precursors in green. This movie highlights behavior at the wavefront. Frames from this movie are shown in Fig. 5e.

Title: Supplementary Movie 10

Description: Neuron-glia precursor interactions in the developing cochlea. A movie from a ~E14-E14.5 Neurog1CreERT2; Ai14; PLP-GFP cochlea, with tdTomato+ SGN peripheral processes in red and PLP-GFP+ glial precursors in green. This movie highlights behavior behind the wavefront. Frames from this movie are shown in Fig. 5f.

Title: Supplementary Movie 11

Description: Neuron-glia precursor interactions in the developing cochlea. A movie from a ~E14-E14.5 Neurog1CreERT2; Ai14; PLP-GFP cochlea, with tdTomato+ SGN peripheral processes in red and PLP-GFP+ glial precursors in green. This movie highlights behavior behind the wavefront. Frames from this movie are shown in Fig. 5g.
