# Supplementary material for: Neuronal processes and glial precursors form a scaffold for wiring the developing mouse cochlea
Source: Nat Commun. 2020 Nov 17;11:5866. doi: 10.1038/s41467-020-19521-2 (PMC7672226; doi:10.1038/s41467-020-19521-2)
Supplement: Supplementary file 15 — Reporting Summary [file 41467_2020_19521_MOESM15_ESM.pdf]

nature research

Corresponding author(s): Lisa Goodrich

Last updated by author(s): Sep 14, 2020

Reporting Summary

Nature Research wishes to improve the reproducibility of the work that we publish. This form provides structure for consistency and transparency in reporting. For further information on Nature Research policies, see our Editorial Policies and the Editorial Policy Checklist.

Statistics

For all statistical analyses, confirm that the following items are present in the figure legend, table legend, main text, or Methods section.

n/a	Confirmed	
☐	☒	The exact sample size (n) for each experimental group/condition, given as a discrete number and unit of measurement
☐	☒	A statement on whether measurements were taken from distinct samples or whether the same sample was measured repeatedly
☐	☒	The statistical test(s) used AND whether they are one- or two-sided<br>*Only common tests should be described solely by name; describe more complex techniques in the Methods section.*
☐	☒	A description of all covariates tested
☐	☒	A description of any assumptions or corrections, such as tests of normality and adjustment for multiple comparisons
☐	☒	A full description of the statistical parameters including central tendency (e.g. means) or other basic estimates (e.g. regression coefficient) AND variation (e.g. standard deviation) or associated estimates of uncertainty (e.g. confidence intervals)
☐	☒	For null hypothesis testing, the test statistic (e.g. F, t, r) with confidence intervals, effect sizes, degrees of freedom and P value noted<br>*Give P values as exact values whenever suitable.*
☒	☐	For Bayesian analysis, information on the choice of priors and Markov chain Monte Carlo settings
☒	☐	For hierarchical and complex designs, identification of the appropriate level for tests and full reporting of outcomes
☒	☐	Estimates of effect sizes (e.g. Cohen's d, Pearson's r), indicating how they were calculated

Our web collection on statistics for biologists contains articles on many of the points above.

Software and code

Policy information about availability of computer code

Data collection	No custom software was used. All data were collected using Imaris 8.1.
Data analysis	Data were analyzed using Prism 8.0 (Graphpad), Metamorph 7.8, and ImageJ FIJI 1.5a.

For manuscripts utilizing custom algorithms or software that are central to the research but not yet described in published literature, software must be made available to editors and reviewers. We strongly encourage code deposition in a community repository (e.g. GitHub). See the Nature Research guidelines for submitting code & software for further information.

Data

Policy information about availability of data

All manuscripts must include a data availability statement. This statement should provide the following information, where applicable:

- Accession codes, unique identifiers, or web links for publicly available datasets
- A list of figures that have associated raw data
- A description of any restrictions on data availability

A data availability statement is provided in the manuscript. This statement indicates that "Projections of the original reconstructed cochleae, examples of movies used to collect data, and the source data for graphs in Figures 1 to 5 are provided with this paper. The relevant statistical analyses are summarized in Supplemental Table 1. For all quantifications, the raw data are shown along with means and standard errors of the means. The original images and movies used to generate these data are available from the corresponding author upon request."

Field-specific reporting

Please select the one below that is the best fit for your research. If you are not sure, read the appropriate sections before making your selection.

☒ Life sciences ☐ Behavioural & social sciences ☐ Ecological, evolutionary & environmental sciences

For a reference copy of the document with all sections, see nature.com/documents/nr-reporting-summary-flat.pdf

Life sciences study design

All studies must disclose on these points even when the disclosure is negative.

Sample size	Sample sizes were selected based on published work from our laboratory and others, which show that a sample size of 3 cochleae in each condition is sufficient. For each cochlea, we analyzed all cells that could be confidently reconstructed or scored, as outlined in the Methods.<br><br>The highly stereotyped development of SGNs between cochlea and the stereotyped migratory properties between adjacent cells, combined with the effect size, the low variability between groups, and the limits of of cell migratory rates support use of 3 biological replicates. See for example J. Neurosci.35, p16221–35 (2015) and J. Neurosci.33,3679–91 (2013).<br><br>This is in line with other ex vivo live imaging and cell tracking studies that show 3 or sometimes fewer replicates. See for example: Nature Communications volume 11, Article number: 3231 (2020), Nature Communications volume 10, Article number: 2859 (2019), Nature Communications volume 10, Article number: 3178 (2019), Nature Communications volume 9, Article number: 4144 (2018), Nature Communications volume 8, Article number: 649 (2017), Nature Communications volume 7, Article number: 13865 (2016), Nature Communications volume 6, Article number: 8497 (2015), and J Biol Chem . 2018 Jul 3;293(28):11143-11153; PNAS April 22, 2014 111 (16) 5896-5901).<br><br>Please also see: https://www.nature.com/articles/nmeth.3091?draft=collection.
Data exclusions	One cochlea was excluded from the analysis in Figure 2 due to incomplete labeling that prevented an unbiased assessment of morphology. For time lapse imaging analysis shown in Figure 3, only trajectories with at least 4 timepoints were included, so that we could be confident that the behavior in that time frame was likely to be reflective of behavior over longer time frames. These were not pre-established criteria.
Replication	Replication was achieved by analyzing SGNs in different cochleae. All experiments were performed independently. All replication attempts were successful. The number of replicates for each experiment is listed in Supplementary Table 1.
Randomization	No experimental groups were used in this study. Each set of cochleae was collected for a specific experiment and all cochleae of the correct genotype were used.
Blinding	SGN reconstructions were analyzed by an independent investigator who was blind to possible outcomes. It was not possible to blind analysis of the trajectories, as the position of each neurite and its behavior and relationship to surrounding glial precursors is obvious when viewing the movie and performing the analysis. However, the data were collected without any outcome in mind.

Reporting for specific materials, systems and methods

We require information from authors about some types of materials, experimental systems and methods used in many studies. Here, indicate whether each material, system or method listed is relevant to your study. If you are not sure if a list item applies to your research, read the appropriate section before selecting a response.

Materials & experimental systems

n/a	Involved in the study	
☐	☒	Antibodies
☒	☐	Eukaryotic cell lines
☒	☐	Palaeontology and archaeology
☐	☒	Animals and other organisms
☒	☐	Human research participants
☒	☐	Clinical data
☒	☐	Dual use research of concern

Methods

n/a	Involved in the study	
☒	☐	ChIP-seq
☒	☐	Flow cytometry
☒	☐	MRI-based neuroimaging

Antibodies

Antibodies used	rabbit anti-DsRed (Clontech, 632496; 1:1000), Rabbit anti-Tuj (Biolegend, PRB-435P-100, 1:1000), Goat anti-GFP FITC (Abcam, AB6662, 1:500), Chicken anti-Neurofilament (Millipore, AB5539, 1:1000), Chicken anti-GFP (Aves, GFP-1020, 1:2000), Donkey anti-Goat A488 (Thermo Fisher, A-11055, 1:500), Donkey anti-Chicken A488 (Jackson ImmunoResearch, 703-545-155, 1:500), Donkey anti-Rabbit A488 (Jackson ImmunoResearch, 711-545-152, 1:500), Donkey anti-Rabbit A594 (Jackson ImmunoResearch, 711-585-152,

	1:500).
Validation	Labeling with anti-dsRed and anti-GFP was validated by comparison with the endogenous tdTomato and GFP signal in the cochlea. Labeling with anti-Tuj and anti-Neurofilament was validated by comparison with published results and our experience using these antibodies in the past to characterize cochlear wiring patterns. All of these antibodies are used standardly across thousands of research laboratories. No new antibodies were used nor does this study demonstrate any novel staining patterns.

Animals and other organisms

Policy information about studies involving animals; ARRIVE guidelines recommended for reporting animal research

Laboratory animals	Mice were housed under a 12 hour/10 hour light/dark cycle at 18-23 C with 40-60 % humidity.<br><br>This study used mice of the following strains, genotype, and sex:<br>Bhlhb5(Cre);Ai14 males on a mixed background, with embryos collected from CD1 females (Charles River Laboratories)<br>Neurog1(CreERT2);Ai14 males on a mixed background, with embryos collected from a CD1 female (Charles River Laboratories)<br>PLP-GFP males either carrying Neurog1(CreERT2);Ai14 or on their own and then crossed to CD1 females (Charles River Laboratories)<br><br>The papers describing each transgenic strain are referenced in the Methods section of the manuscript.<br>All of these animals were bred as adults (>6 weeks old).<br>Embryos were collected from pregnant dams between ~E14 and E16.
Wild animals	No wild animals were used for this research.
Field-collected samples	This study did not involve samples collected from the field.
Ethics oversight	Animals were maintained and handled according to a protocol approved by the I.A.C.U.C. at Harvard Medical School.

Note that full information on the approval of the study protocol must also be provided in the manuscript.
